# Editorial

**Published:** 1876-10

**Authors:** 


### Editorial.

### THE CHICAGO BOTANICAL GARDEN.

Australia, which was peopled in the year 1870 by less than one million of inhabitants, can point to five botanical gardens established within its borders. There are five also in South America. That in Calcutta is more than one hundred years old, and covers two hundred and sixty acres of ground. Java possesses in the Buitenzorg, probably the largest garden in the world, as it includes two hundred and seventy-two acres of ground, and furnishes sites for plant culture at elevations from 4,500 to 10,000 feet above the level of the sea. Up to within a recent date there was but one botanical garden in North

America — that connected with the University of Harvard. We say but one, for it is well known that the Government gardens at Washington subserve chiefly the purpose of providing bouquets for members of Congress, while that at St. Louis — small, popular and showy — is scarcely adapted to the study of botanical science. The Cambridge garden, now seventy-five years old, is completely filled with collections obtained from the botanists of the United States; but its chief usefulness has been to enable Professor Gray to advance his botanical researches. It covers ten acres of ground, and to this space has been recently added the ground for an arboretum.

The Chicago Botanical Garden has been called into existence so noiselessly, and has accomplished its work thus far so unpretentiously, that there are many who will first learn of its existence from a perusal of these pages. Curiously enough, the largest number of those who are already familiar with its aim and labors, are residents in foreign lands.

The Chicago garden was created by the action of the South Park Commissioners, who organized a Board of Management, consisting of Judge H. N. Hibbard, Gov. Wm. Bross, and Messrs. E. H. Sargent and Albert E. Ebert. Prof. H. H. Babcock, the eminent Chicago botanist, was appointed Director. The garden is located in the southeast corner of the northern and western of the two south parks. Fifteen acres are allotted for the garden proper, which now contain plants arranged in their natural order; and sixty additional adjacent acres have been assured for further use. Three-fourths of this latter space will be occupied as an arboretum, in which the pines, oaks, maples, etc., will be separately arranged; and special provision will be made for medicinal plants, which will be also systematically grouped.

It is safe to say that a botanical garden was never before established which has, in a similar space of time, accomplished the results which the management now actually exhibit. Since the date of organization, on the 10th of April, 1875, 8,000 packets of seeds and living plants have been received here from England, France, Germany, Russia, Holland, Italy, Austria, Hungary, Java, Australia, Chili, South Africa (Cape of Good

Hope), Calcutta, and various portions of the United States, the latter chiefly from the garden at Cambridge. These represent about 6,000 living species. During this same time, also, about 2,000 packets of living plants and seeds have been exported to various parts of the world, principally in exchange to the countries mentioned above. It may be added that these exchanges have been eagerly sought for abroad, and that nothing could exceed the cordial and even enthusiastic support accorded to the Chicago enterprise, by strangers who have proved themselves friends in these remote quarters of the globe. As an illustration of this fact, it may be mentioned that it has actually been found to be more difficult to obtain collections from various parts of this country than from Java and South Africa.

To-day the four houses, heated by warm water, and so arranged that the temperature in each may be adjusted to a degree, are crowded to their utmost capacity. Many tropical plants have reached the glass roofs, and turned downward in accordance with the demands of a vigorous growth. Specimens of the *Eucalyptus Globulus*, grown from Australian seed twelve months ago, are now fully twelve feet in height. Among the medicinal plants to be seen, there are several varieties of hyosciamus, aconite and arnica.

This success has been largely due to the indefatigable labors of Prof. Babcock, who, prior to the inauguration of the scheme, dispatched 15,000 herbaceous specimens to Java, Belgium, Calcutta, Australia, Sicily and Chili; and the garden was not slow in reaping the reward of this generous insemination. The Board of Management were also fortunate in securing the services of a skilled gardener, who had served five years in the botanical garden at Kew, and had also been for five years in charge of the garden at Belfast, in Ireland.

A register of visitors, kept upon the grounds, records at present 327 names, representing a large number of gentlemen from outside of the city — those from Boston, Philadelphia, New York and Washington preponderating.

Our object in thus prominently calling attention to the Chicago garden is to aid in furthering the judicious aims of the Board of Managers. The ultimate and permanent success of their scheme will depend largely upon the success which they achieve in awaking not only a popular interest in the botanical garden, which is eminently desirable, but also in securing the moral support and encouragement of men of culture and science in the city and its environs. Such success will involve the development, among the masses of our people, of a taste for horticulture and arboriculture, as well as of a tendency to seek that highest form of recreation from the cares of business, which is afforded by the direction of the mind to the practical study of natural science. In England and elsewhere, it is not rare to find clergymen, barristers and gentlemen eminent in various professions and stations of life, resorting to this class of studies as a source of recreation and amusement, and often they attain a truly remarkable degree of proficiency in their favorite science. Such a state of society will obtain more and more in this country, as capital accumulates its surplus, and the means of generous living are placed at the disposal of men in the middle ranks of life. In the territory around Chicago — a city destined without doubt to be the metropolis of a continent, its feet resting on a soil which is a treasure house of vegetable and mineral wealth, its arms reaching out to the embrace of an empire of unparalleled productive resources — such conditions as those described will inevitably arrive.

Meantime, we believe that we repeat the sentiment of the medical profession of our city in expressing the hope, that the South Park Commissioners will continue to support and extend the work of the Chicago Botanical Garden, in the liberal and far-seeing spirit with which it has been projected and thus far developed.

